# Supplementary material for: Chromatin topology control by a muscle-specific ribosomal protein
Source: bioRxiv. 2026 Jun 26:2026.06.23.733628. Preprint. [Version 1] doi: 10.64898/2026.06.23.733628 (PMC13320957; doi:10.64898/2026.06.23.733628)
Supplement: Supplement 1 [file NIHPP2026.06.23.733628v1-supplement-1.pdf]

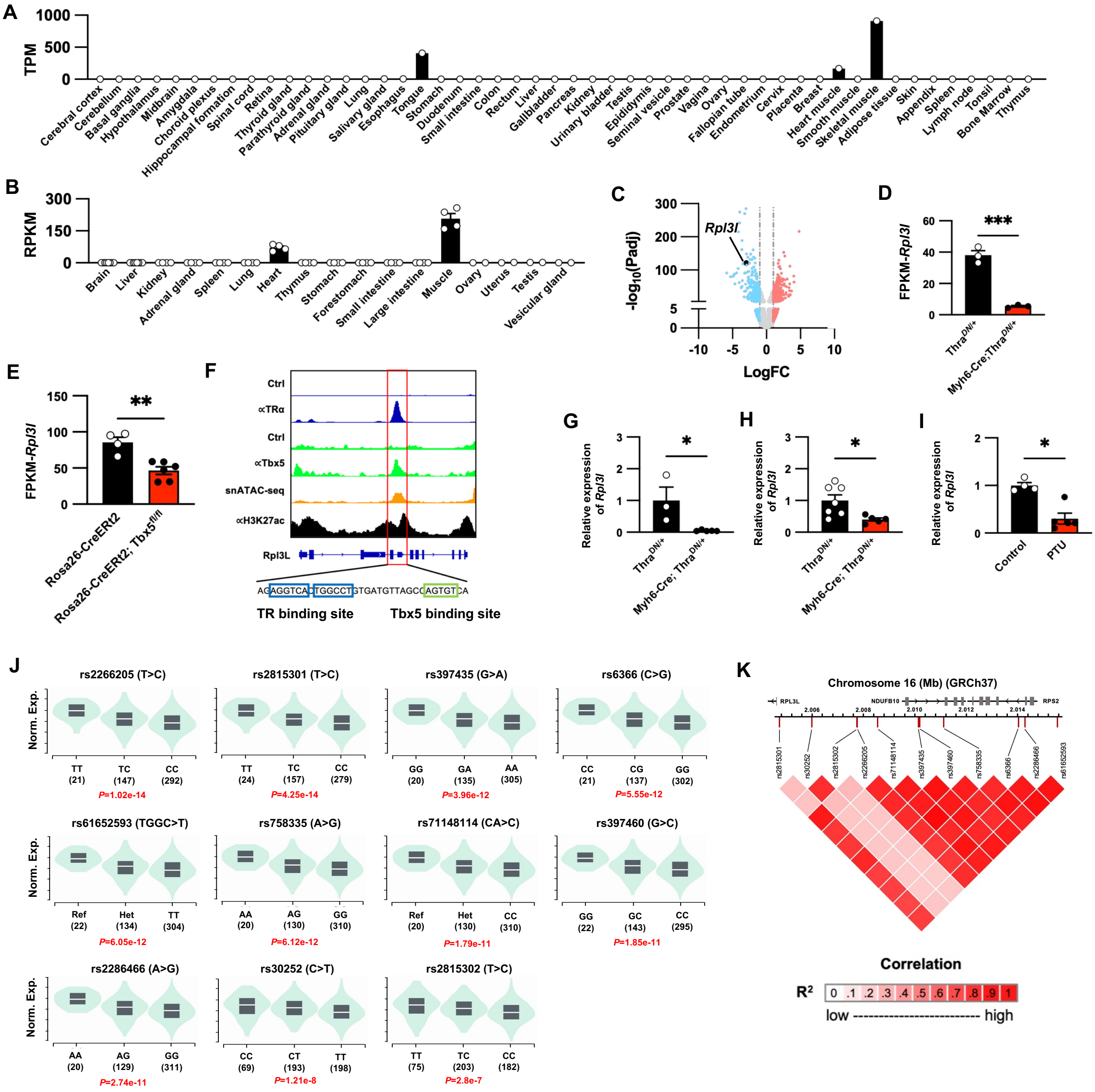

Supplementary Figure 1. Thyroid hormone signaling and atrial fibrillation–associated genetic variation converge on *Rpl3l* regulation

(A) Tissue distribution of *RPL3L* expression across human organs from RNA-seq datasets, shown as transcripts per million (TPM).

(B) Tissue distribution of *Rpl3l* expression across mouse organs from RNA-seq datasets, shown as reads per kilobase per million mapped reads (RPKM).

(C) Volcano plot showing differential gene expression in *Myh6*-Cre; *Thra*$^{DN/+}$ hearts.

(D) *Rpl3l* expression in ventricles from *Thra*$^{DN/+}$ and *Myh6*-Cre; *Thra*$^{DN/+}$ mice.

(E) *Rpl3l* expression in ventricles from Rosa26^{CreERT2} control and Rosa26^{CreERT2}; *Tbx5*$^{fl/fl}$ mice.

(F) Genome browser tracks showing TRα, Tbx5, H3K27ac ChIP-seq, and snATAC-seq signal at the *Rpl3l* locus. The predicted TR and Tbx5 binding sites are indicated below the tracks.

(G-H) RT-qPCR analysis of *Rpl3l* expression in P14 (G) and adult (H) left atria from *Thra*$^{DN/+}$ and *Myh6*-Cre; *Thra*$^{DN/+}$ mice.

(I) RT-qPCR analysis of *Rpl3l* expression in adult left atria from control and PTU treated mice.

(J) Distribution of *RPL3L* expression in human atrial appendage tissue from GTEx, stratified by genotype for atrial fibrillation–associated variants. Normalized expression is shown for each genotype group; nominal *P* values are indicated.

(K) Linkage disequilibrium structure among variants associated with reduced *RPL3L* expression in human atrial appendage tissue. Pairwise correlation is shown as R^2.

Data are shown as mean ± SEM. Each dot represents an individual animal. *$P < 0.05$, **$P < 0.01$, and ***$P < 0.001$

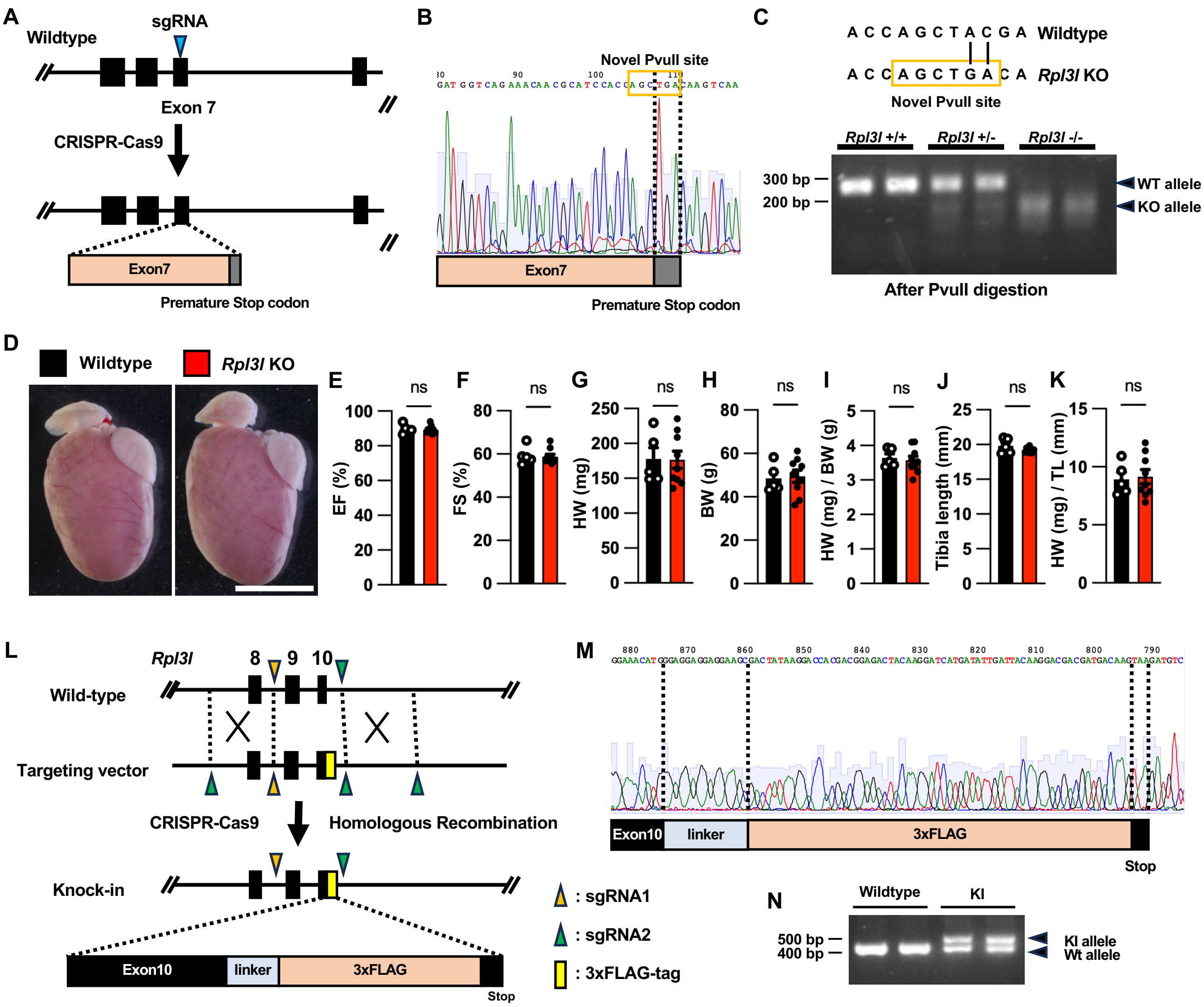

Supplementary Figure 2. Generation and phenotypic validation of *Rpl3l* KO and *Rpl3l*^{FLAG} mice

(A) Schematic of the CRISPR-Cas9 genome-editing strategy used to generate *Rpl3l* KO mice. The sgRNA was designed to target exon 7 of the *Rpl3l* locus, and homology-directed repair introduced a premature stop codon within exon 7.

(B) Sanger sequencing chromatogram confirming introduction of the engineered mutant allele. The mutation introduced a premature stop codon together with a novel PvuII restriction site, CAG^CTG, within exon 7.

(C) PCR-based genotyping strategy using PvuII restriction digestion. Wild-type mice show the undigested allele, heterozygous mice show both wild-type and digested mutant alleles, and homozygous *Rpl3l* KO mice show only the digested mutant allele, confirming germline transmission of the edited allele.

(D) Representative gross heart images from wildtype and *Rpl3l* KO mice.

(E–K) Quantification of baseline cardiac anatomical and functional parameters in wildtype and *Rpl3l* KO mice, including ejection fraction (EF) (E), fractional shortening (FS) (F), heart weight (HW) (G), body weight (BW) (H), heart weight normalized to body weight (HW/BW) (I), tibia length (J), and heart weight normalized to tibia length (HW/TL) (K).

(L) Schematic of the CRISPR-Cas9 mediated knock-in strategy used to generate C-terminally tagged *Rpl3l*^{FLAG} mouse. sgRNAs were designed to target the region surrounding exon 10 of the *Rpl3l* locus, and homologous recombination introduced a linker sequence followed by a 3 × FLAG tag immediately downstream of exon 10.

(M) Sanger sequencing chromatogram confirming correct insertion of the linker-3 × FLAG cassette at the C terminus of *Rpl3l*. The exon 10, linker, 3 × FLAG, and stop-codon regions are indicated.

(N) PCR-based genotyping of wildtype and *Rpl3l*^{FLAG} mice. Wildtype mice show the unmodified allele (400 bp), whereas *Rpl3l*^{FLAG} mice show both the wildtype and knock-in alleles (500 bp), confirming successful germline transmission of the targeted allele.

Data are shown as mean ± SEM. Each dot represents an individual animal. Scale bar, 1 cm. ns, not significant

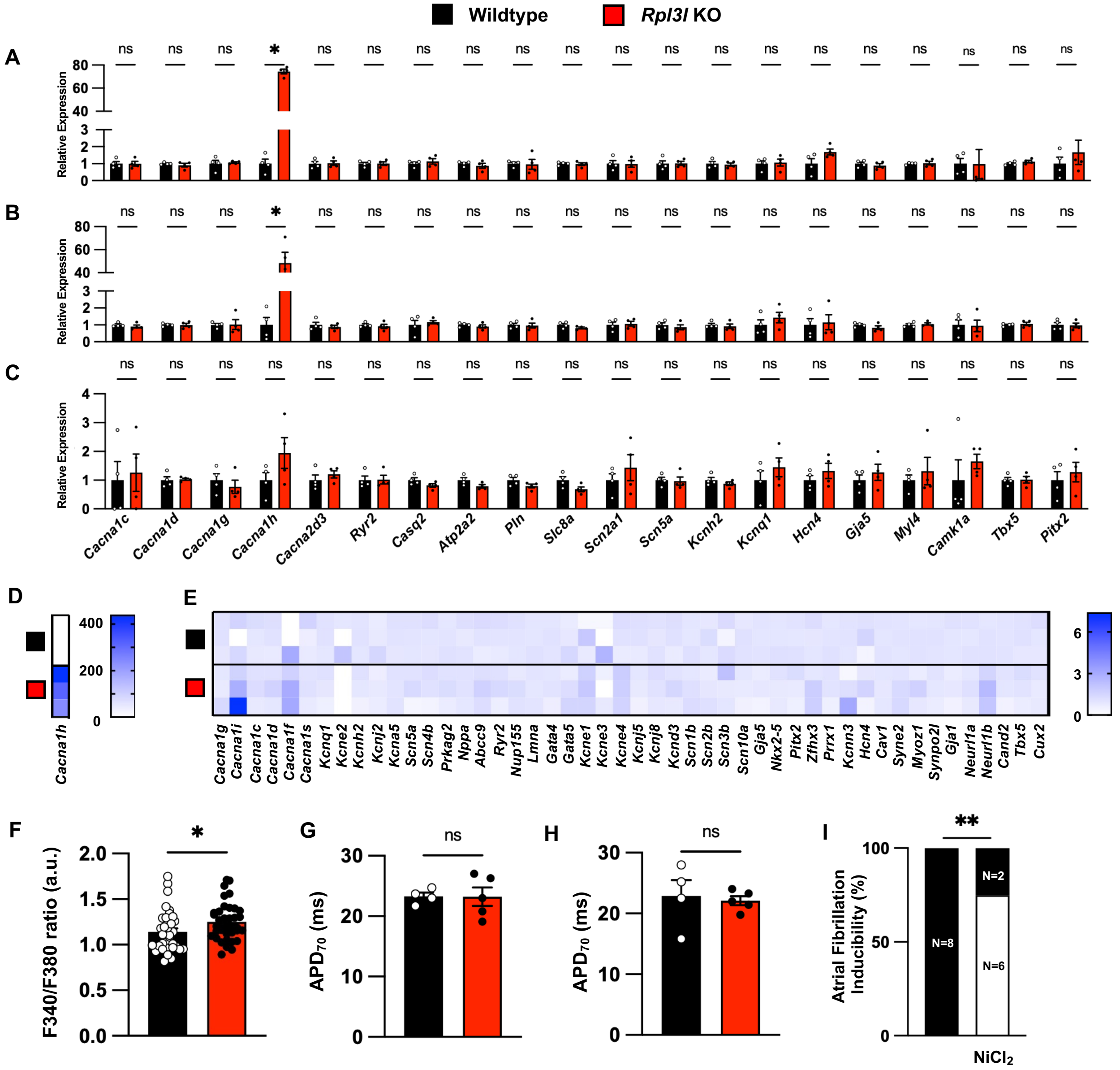

Supplementary Figure 3. *Rpl3l* deficiency selectively derepresses *Cacna1h* and disrupts atrial Ca^{2+} homeostasis.

(A – C) RT-qPCR analysis of selected cardiac ion-channel and calcium-handling genes in mouse wildtype and *Rpl3l* KO right atria (A), left atria (B), and ventricle (C).

(D and E) Summary heatmaps showing relative expression of *Cacna1h* (D) and additional ion-channel and calcium signaling pathway genes (E) in wildtype and *Rpl3l* KO atrial samples. Color scales indicate relative expression.

(F) Quantification of the F340/F380 ratio metric Ca^{2+} signal in wildtype and *Rpl3l* KO atria. Each dot represents an individual atrial cardiomyocyte.

(G and H) Quantification of atrial action potential duration at 70% repolarization (APD_{70}) following KN-93 treatment in right atria (G) and left atria (H) from wildtype and *Rpl3l* KO mice.

(I) Quantification of AF inducibility with $NiCl_2$. Numbers within bars indicate the number of animals in each category shown in Figure 1E.

Data are shown as mean ± SEM. Each dot represents an individual animal. ns, not significant; $^*P < 0.05$, and $^{**}P < 0.01$.

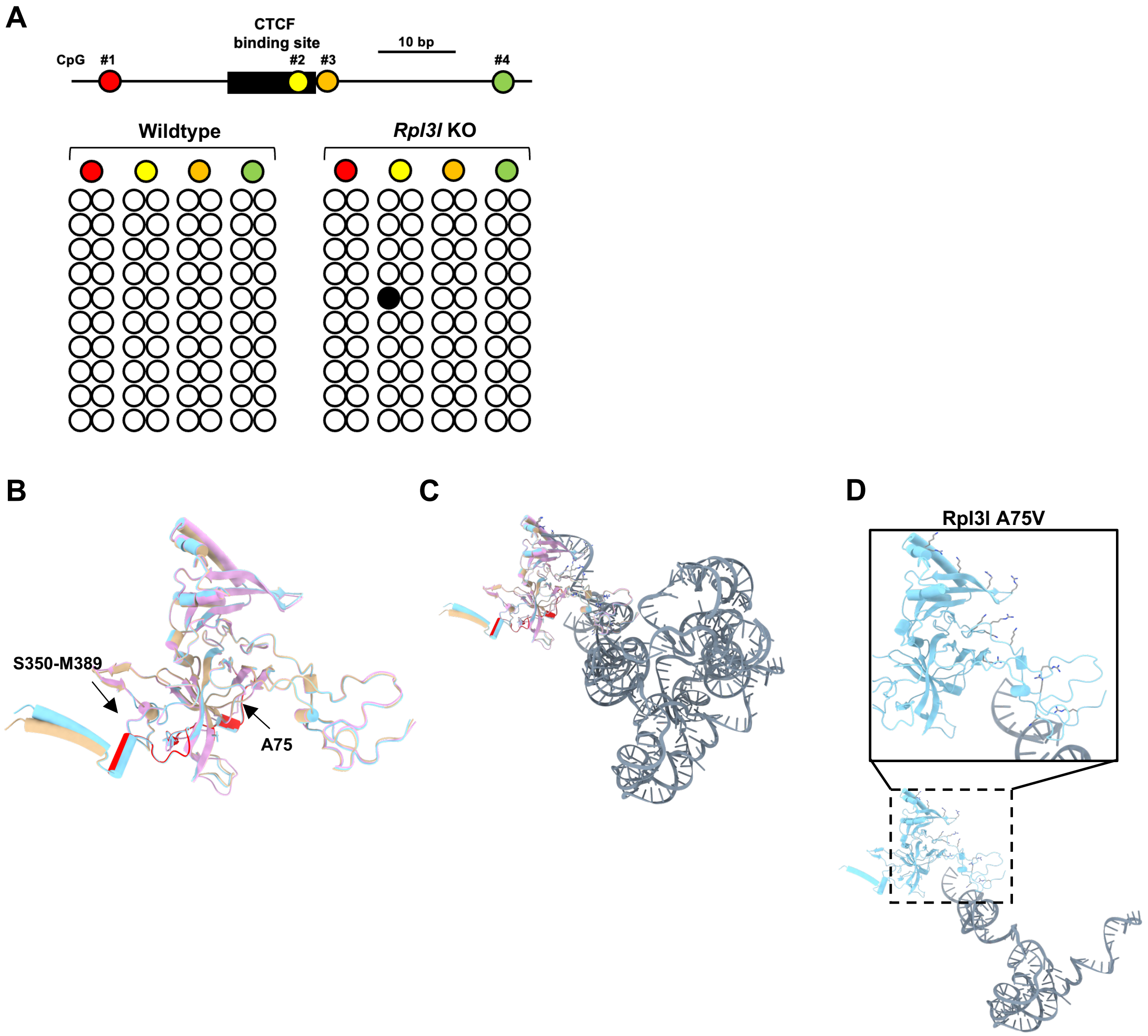

Supplementary Figure 4. Molecular characterization of Rpl3l mutants and predicted RNA-associated structural features
(A) Bisulfite sequencing analysis of CpG methylation across the predicted CTCF-binding region. The schematic indicates the positions of CpG sites analyzed relative to the CTCF-binding site. Open circles represent unmethylated CpGs, and filled circles represent methylated CpGs in individual sequenced clones from wild-type and *Rpl3l* KO samples.
(B) Structural alignment of predicted Rpl3l WT, Rpl3l A75V, and Rpl3l ΔS350–M389 models. WT, A75V, and ΔS350–M389 models are shown in tan, cyan, and magenta, respectively. The A75 residue and the S350–M389 region are highlighted in red.
(C) Overview of the predicted Rpl3l structural model aligned to an RNA-containing ribosomal structural context. Rpl3l is shown in the aligned protein model colors, and RNA is shown in gray, illustrating the predicted spatial relationship between Rpl3l and nearby RNA.
(D) AlphaFold predicted structural models showing RNA-proximal basic residues in Rpl3l A75V mutant.
